# Cost-effectiveness of iruplinalkib versus crizotinib in first-line anaplastic lymphoma kinase-positive advanced non-small-cell lung cancer patients in China

**DOI:** 10.3389/fphar.2025.1651463

**Published:** 2025-10-20

**Authors:** Wanjie Zhang, Yuqiong Lu, Zhanjing Dai, Linning Wang, Yang Zhou, Yun Lu, Feng Chang

**Affiliations:** School of International Pharmaceutical Business, China Pharmaceutical University, Nanjing, China

**Keywords:** iruplinalkib, cost-effectiveness, non-small cell lung cancer, China, crizotinib, anaplastic lymphoma kinase

## Abstract

**Background:**

The recently completed INSPIRE trial demonstrated that iruplinalkib improved progression-free survival and intracranial antitumor activity compared with crizotinib in patients with anaplastic lymphoma kinase (ALK) -positive non-small-cell lung cancer (NSCLC). The objective of this study was to determine the potential cost-effectiveness of iruplinalkib vs. crizotinib in the Chinese healthcare setting.

**Methods:**

A cost-effectiveness model was developed using the partition survival method, with three health states: progression-free survival, progressive disease, and death. Data from the INSPIRE trial were used to estimate progression-free and overall survival. Costs included drug treatment, disease management, and adverse events management. Drug costs and utilities were the main drivers of the model in the deterministic sensitivity analysis.

**Results:**

Treatment with iruplinalkib versus crizotinib resulted in a gain of 0.55 life-years, 2.11 quality-adjusted life-years (QALYs), and an incremental cost of $4,325.55, resulting in an incremental cost-effectiveness ratio of $2,048.03/QALY. Drug costs and utilities were the main drivers of the model in the deterministic sensitivity analysis. From the probabilistic sensitivity analysis (PSA), iruplinalkib had a 100% probability of being cost-effective at a willingness-to-pay threshold of $13,447.89/QALY.

**Conclusion:**

Compared to crizotinib, iruplinalkib is a cost-effective therapy for treatment-naïve patients with ALK-positive NSCLC.

## 1 Introduction

Lung cancer ranks first both in incidence and mortality worldwide, with the highest incidence and mortality rates observed in China (Bray et al., 2024; Zheng et al., 2024). Non-small cell lung cancer (NSCLC) is the predominant subtype of lung cancer, accounting for approximately 85% (Oser et al., 2015). Currently, the survival rate for patients with end-stage disease (IIIB/IV) remains poor, with a 5-year relative survival rate of only 5.8% for those with distant metastases (National Cancer Institute, 2020). With the continued identification and in-depth research of lung cancer pathogenic genes, molecular-targeted therapy has become the primary approach to improving the prognosis for NSCLC patients (Guo et al., 2022).

Anaplastic lymphoma kinase (ALK) is a potent oncogenic driver in lung cancer (Schneider et al., 2023). ALK gene rearrangements are detected in 3%–5% of NSCLC cases (Huang et al., 2020). Targeted therapy with tyrosine kinase inhibitors (TKIs) has shown greater clinical improvement than conventional chemotherapy in patients with ALK rearrangements (Khambata-Ford et al., 2010; Pirker et al., 2009; Solomon et al., 2014). According to clinical guidelines from the European Society for Medical Oncology (ESMO), the National Comprehensive Cancer Network Clinical Practice Guidelines in Oncology (NCCN), and Guidelines of the Chinese Society of Clinical Oncology (CSCO) for NSCLC, first-line treatment for patients with ALK gene mutations includes ceritinib, alectinib, brigatinib, lorlatinib, and these are all prioritized (Lee et al., 2024; Riely et al., 2024; Medical Oncology Branch of China International Exchange and Promotive Association for Medical and Health Care, 2024). Several pharmacoeconomic studies have compared these agents with crizotinib across diverse clinical and economic settings (Loong et al., 2020; Carlson et al., 2018; Cr et al., 2022; Li et al., 2021). Nevertheless, there is currently a lack of cost-effectiveness evidence assessing the use of iruplinalkib as a first-line therapy for the same indication.Crizotinib, the first targeted TKI for advanced ALK-positive (ALK+) NSCLC, was approved by the US Food and Drug Administration (FDA) in 2011 (FDA, 2023) and by the China National Medical Products Administration (NMPA) in 2013 (Liu et al., 2019). Crizotinib remains the standard treatment for previously untreated patients with ALK + NSCLC (Riely et al., 2024; China clinical practice guideline for stage Ⅳ primary lung cancerChinese Association for Clinical Oncologists, 2024). However, crizotinib has been associated with challenges, including drug resistance and suboptimal efficacy in patients with brain metastases (Katayama et al., 2012; Casaluce et al., 2016). Therefore, novel ALK-TKIs are needed to address these challenges.

Iruplinalkib, a new-generation drug, was approved by NMPA in 2023 to treat crizotinib-resistant or intolerant, locally advanced, metastatic ALK + NSCLC (Yang et al., 2023). Its long-term clinical benefits and cost-effectiveness have been widely recognized, resulting in its inclusion in the 2023 National Reimbursement Drug List (NRDL). In 2024, iruplinalkib was further approved by NMPA for the treatment of patients with ALK + NSCLC who previously had not received ALK-TKI treatment. The randomized, open-label, Phase III INSPIRE trial demonstrated that crizotinib-naïve ALK + patients treated with iruplinalkib had significantly longer progression-free survival (PFS) (median PFS, 27.7 months [95% CI, 26.3-NE]) compared to patients treated with crizotinib (median PFS, 14.6 months [95% CI, 11.1-16.5]). Iruplinalkib also showed a significantly lower hazard ratio for disease progression or death (0.34, [98.02%CI, 0.23-0.52]; p < 0.0001) (Shi et al., 2024). The trial also demonstrated that iruplinalkib was more effective in patients with central nervous system (CNS) metastases. The intracranial objective response rate (ORR) was significantly higher in the iruplinalkib group (90.9%, 95% CI, 58.7-99.8) than in the crizotinib group (60.0%, 95% CI, 32.3-83.7).

Economic considerations have become a key indicator of whether a drug will be included in the NRDL and formulary. Although iruplinalkib showed superior efficacy over crizotinib in the INSPIRE trial, the economic impact and value of iruplinalkib in the first-line setting have not been evaluated. According to the comparator selection recommendations in the China Guidelines for Pharmacoeconomics Evaluation 2020 (Liu et al., 2020), crizotinib was selected as a suitable comparator because of the same indication, and availability of head-to-head evidence versus iruplinalkib. Therefore, this study aims to evaluate the cost-effectiveness of iruplinalkib versus crizotinib in treatment-naïve ALK + NSCLC patients from the perspective of the Chinese healthcare system, based on the INSPIRE clinical trial.

## 2 Methods

### 2.1 Model structure

**FIGURE 1:**
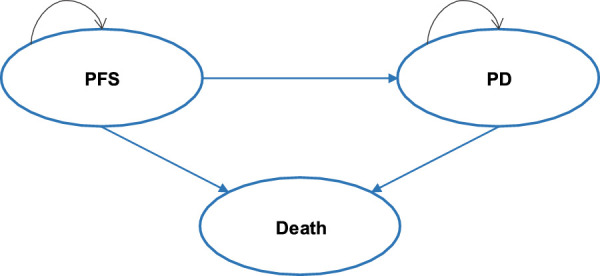
Model structure. PFS, progression-free survival; PD, progression disease.

The cost-effectiveness model was developed using Microsoft Excel^®^ (Redmond, WA, United States of America) with three health states: progression-free survival (PFS), progressive disease (PD), and death (see Figure 1). A partitioned survival model (PSM) was developed to simulate disease progression in patients with advanced ALK + NSCLC who had not previously received systemic therapy. The distribution of patients across different health states over time was estimated by calculating the area under the survival curve. At each cycle, patients may either remain in the previous state, progress to a disease progression state, or enter the death state, also known as the absorption state.

Patients received iruplinalkib (60 mg once daily for the first 7 days, followed by an increased dosage of 180 mg once daily thereafter) or crizotinib (250 mg twice daily) until disease progression. Subsequent treatment strategies were guided by clinical guidelines and medication data collected in the INSPIRE trial. The model was conducted with 4-week cycles over a 25-year horizon to approximate a lifetime perspective. Patients in the INSPIRE trial entered the model at a mean age of 55 years; thus, the horizon extends to approximately 80 years, exceeding the 2021 Chinese life expectancy (78.2 years). By the end of the model, cumulative mortality of both groups was approximately 90% (iruplinalkib) and 93% (crizotinib), indicating negligible residual survival. Therefore, extending the time horizon further would have minimal impact on model outcomes. In the base-case analysis, both costs and utilities were discounted at 5% annually, as recommended by the China Guidelines for Pharmacoeconomic Evaluation (Liu et al., 2020). Costs from previous years were adjusted using the Consumer Price Index (CPI) and reported in 2024 USD dollars (1 USD = 7.12 CNY). The primary outcomes of this study included costs, quality-adjusted life years (QALYs), life years (LYs), and incremental cost-effectiveness ratios (ICERs). The willingness-to-pay (WTP) threshold was defined as 1-3 times the *per capita* Gross Domestic Product (GDP) of China in 2024, estimated at $13,452.37 (¥95,749). Additionally, a treatment is considered highly cost-effective if the ICER is less than one times the GDP *per capita*.

### 2.2 Clinical inputs

**FIGURE 2:**
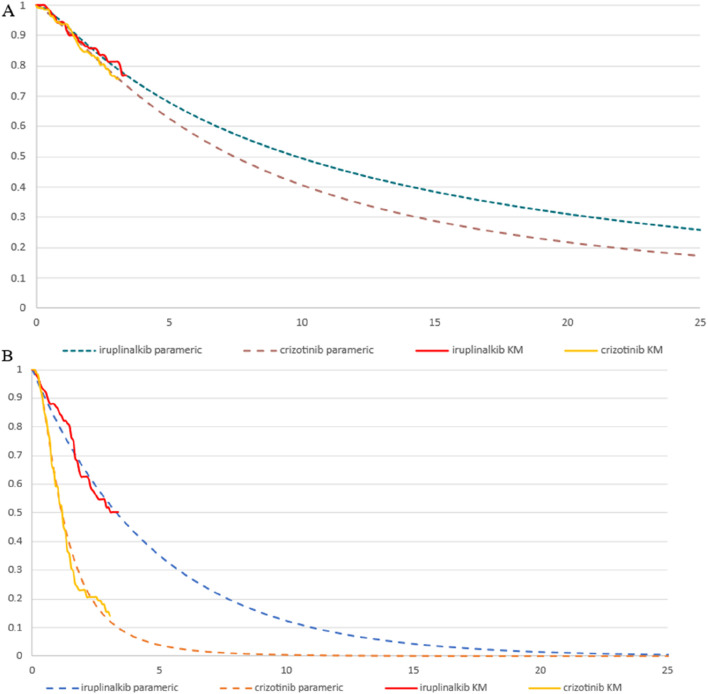
Parametric survival curve fits the Kaplan-Meier (KM) trial data **(A)** exponential overall survival curve fits to the KM trial data **(B)** log-logistic progression-free survival curve fits to the KM trial data.

The PFS and overall survival (OS) data for iruplinalkib and crizotinib were obtained from the INSPIRE study (Shi et al., 2024). Parametric survival functions were fitted to the Kaplan-Meier data assessed by an independent review committee (Latimer, 2011). Based on the clinical rationale, visual fit, and statistical goodness-of-fit [Akaike information criterion (AIC) and Bayesian information criterion (BIC)], the log-logistic distribution was more appropriate for the PFS curve to fit for iruplinalkib and crizotinib. Although the AIC and BIC of crizotinib indicated that the log-normal distribution was the most appropriate and the log-logistic distribution was suboptimal, the distribution should remain the same for both groups, as recommended by the National Institute for Health and Care Excellence (NICE) Technical Support Document 14^26^. The exponential distributions were most appropriate for the OS curve to fit for iruplinalkib and crizotinib. (AIC and BIC for PFS and OS are shown in the Electronic Supplementary Material (ESM) Table 1, the parametric survival curve fits are shown in Figure 2). Given that adverse events (AEs) significantly affect both costs and QALYs, their impact was incorporated into the model. Grade 3-5 adverse events and a frequency ≥5.0% in either treatment arm were included (see Table 1). In the cohort analysis, Chinese natural mortality rates were applied to the fitted survival curves to simulate patient survival more accurately (China Population Census Yearbook, 2020, 2020).

**TABLE 1:** Key parameters and their variations.

Parameters	Value	Lower limit	Upper limit	Distribution	Data source
Utility values					
PFS	0.804	0.683	0.925	Beta	Nafees et al. (2017)
PD	0.321	0.273	0.369	Beta	
Disutility of AEs					
Hypertension	−0.040	−0.044	−0.034	Beta	Nafees et al. (2017)
Increased AST levels	−0.037	−0.046	−0.029	Beta	NICE, TA670 (Excellence, 2021)
Increased ALT levels	−0.037	−0.046	−0.029	Beta	
Increased serum CPK levels	−0.037	−0.046	−0.029	Beta	
Abnormal liver function	−0.037	−0.046	−0.029	Beta	
Decrease in ANC	−0.2	−0.18	−0.22	Beta	Nafees et al. (2017)
Drug costs of PFS, per unit, $					
Iruplinalkib (60 mg)	18.33	16.50	20.16	Gamma	The lowest regionally available prices disclosed to the public (Yunnan Healthcare Security Administration, 2025)
Crizotinib (250 mg)	20.32	18.28	22.35	Gamma	MENET database (2025)
Drug costs of PD, per unit, $					
Alectinib(150 mg)	7.99	7.19	8.79	Gamma	
Ensartinib(100 mg)	19.94	17.95	21.94	Gamma	
Ensartinib(25 mg)	6.90	6.21	7.59	Gamma	
Ceritinib(150 mg)	12.57	11.31	13.83	Gamma	
Crizotinib(250 mg)	20.32	18.28	22.35	Gamma	
Iruplinalkib (60 mg)	18.33	16.50	20.16	Gamma	
Cisplatin(30 mg)	2.14	1.06	5.59	Gamma	
Carboplatin(150 mg)	21.95	19.76	24.15	Gamma	
Pemetrexed(200 mg)	153.13	19.76	24.15	Gamma	
Paclitaxel(30 mg)	21.39	19.25	23.53	Gamma	
Bevacizumab(100 mg)	157.63	140.14	210.68	Gamma	
Sintilimab(100 mg)	151.69	136.52	166.86	Gamma	
radiotherapy	6,318.54	5,689.89	6,951.12	Gamma	Clinical opinion
Death, $					
End-of-life care	4,844.15	4,359.73	5,328.56	Gamma	(Li et al., 2018)
AEs cost, $					
Hypertension	14.09	12.68	15.50	Gamma	Wu et al. (2012); Ran et al. (2023) and adjusted by clinical opinion
Increased AST levels	59.36	21.07	65.33	Gamma	
Increased ALT levels	32.12	28.92	35.34	Gamma	
Increased serum CPK levels	7.02	6.32	7.73	Gamma	
Abnormal liver function	40.96	36.85	45.05	Gamma	
Decrease in ANC	14.04	12.64	15.45	Gamma	
Supportive disease management costs, per unit, $					
Outpatient	4.21	3.79	4.64	Gamma	Clinical opinion
Electrocardiogram	2.91	1.12	5.06	Gamma	BHS Administration (2021); GHS Administration (2021); WHS Administration (2021); ZHS Administration (2023); Central Health Services Administration (2021)
Chest CT	43.06	38.76	47.38	Gamma	Lei et al. (2022) and Clinical opinion
Brain CT	43.06	38.76	47.38	Gamma	
Contrast-enhanced CT of the upper abdomen	43.06	38.76	47.38	Gamma	
Contrast-enhanced MRI of the head	123.60	111.24	135.96	Gamma	
Echocardiogram	28.09	25.28	30.90	Gamma	BHS Administration (2021); GHS Administration (2021); WHS Administration (2021); ZHS Administration (2023); Central Health Services Administration (2021)
Complete blood count	2.81	2.11	4.22	Gamma	
Urinalysis	0.24	0	0.42	Gamma	
Stool for routine	0.43	0.23	0.70	Gamma	
D-dimer	4.21	3.79	4.64	Gamma	Clinical opinion
Serum biochemical analysis	21.15	33.78	0.67	Gamma	Clinical opinion
Bone scan	109.68	98.69	120.68	Gamma	Clinical opinion
Tumor markers	77.25	69.54	84.97	Gamma	Clinical opinion
PET-CT	1,025.28	922.75	1,128.93	Gamma	Clinical opinion
Incidence of AEs					INSPIRE trial (Shi et al., 2024)
Hypertension-iruplinalkib	9.10%	——	——	——	
Hypertension- crizotinib	0%	——	——	——	
Increased AST levels-iruplinalkib	6.30%	——	——	——	
Increased AST levels- crizotinib	5.40%	——	——	——	
Increased ALT levels-iruplinalkib	8.40%	——	——	——	
Increased ALT levels- crizotinib	8.10%	——	——	——	
Increased serum CPK levels--iruplinalkib	4.20%	——	——	——	
Increased serum CPK levels--crizotinib	10.70%	——	——	——	
Abnormal liver function-iruplinalkib	9.10%	——	——	——	
Abnormal liver function-crizotinib	3.40%	——	——	——	
Decrease in ANC-iruplinalkib	1.40%	——	——	——	
Decrease in ANC- crizotinib	14.10%	——	——	——	

PFS, progression-free survival; PD, progression disease; AST, aspartate aminotransferase; ALT, alanine aminotransferase; CPK, creatine phosphokinase; ANC, absolute neutrophil count; CT, computed tomography; MRI, magnetic resonance imaging; PET-CT, Positron emission tomography-computed tomography.

### 2.3 Utility inputs

The INSPIRE trial did not include utility measurements; thus, utility values for PFS (0.804) and PD (0.321) were derived from the Chinese subgroup dataset in a study conducted by Nafees et al. (Table 1) (Nafees et al., 2017). Additionally, disutility related to AEs was included in the analysis. Disutility values were obtained from Nafees et al.’s study and a health technology assessment by NICE (see Table 1). In the calculation, the one-time QALY decrements due to AEs were determined by multiplying their disutility values by the corresponding incidence probabilities at the start of the first cycle.

### 2.4 Cost inputs

From the perspective of the Chinese healthcare system, direct costs, including first-line and subsequent-line drugs, disease management across different disease stages, and management of AEs, were incorporated into the model. Treatment costs for each cycle were calculated based on the dosing schedule and unit costs from the MENET database and prices of drugs at local providers (see Table 1; Excellence, 2021; Yunnan Healthcare Security Administration, 2025). The cost of initial drug treatment in the PFS health state was calculated under the assumption that the patients were treated until disease progression or death. Since iruplinalkib and crizotinib are both oral drugs, administration costs were excluded. Costs per cycle in the PD health state were calculated as a weighted aggregate based on subsequent treatment regimens and their respective utilization proportions from the INSPIRE trial (see Table 1). AEs’ costs were calculated once in the first cycle. Most costs were derived from the published literature, and on this basis, opinions of clinical experts in lung cancer were considered (including 38 consultant oncologists specializing in lung cancer treatment from more than 30 grade A tertiary hospitals in 3 Chinese provinces) (see Table 1).

Specifically, the subsequent treatment regimens for the intention-to-treat (ITT) population in clinical trials have been prospectively documented for patients who progressed following first-line treatment. This study hypothesized that the second-line therapeutic agents administered to patients do not overlap with those used in first-line treatment. For example, patients receiving iruplinalkib as a first-line therapy would not receive the same drug in subsequent treatment lines. When processing subsequent-line treatment records, cases were excluded if the same drug was used in both first-line and second-line treatment, if the drugs used in subsequent treatment accounted for less than 1%, or if the subsequent-line drugs had not yet been approved for clinical use. In instances where treatment records did not specify the exact drugs but only described the treatment method (e.g., a combination of targeted therapy and chemotherapy), the cost per cycle of the treatment was calculated by summing the average price of the second-line targeted therapies and the per cycle chemotherapy cost obtained through consultation with clinical experts.

The proportion of patients receiving radiotherapy has been documented in the post-line treatment records of clinical trials. Only a subset of patients receiving chemotherapy after PD also receive concurrent radiotherapy, which overlaps with chemotherapy patients. Therefore, the proportion of patients receiving radiotherapy was calculated separately and not included in the calculation for patients receiving drug treatments. The *per capita* radiotherapy cost was derived from expert consultation. The total cost of radiotherapy in the model was estimated as a one-time value, calculated by multiplying the number of newly progressed patients by the radiotherapy proportion and the average per-patient cost. (see in the ESM Table 2).

**TABLE 2:** The results of base-case analysis.

Interventions	Iruplinalkib	Crizotinib	Difference
PFS			
Drug cost	$84,448.57	$23,137.36	$61,311.21
AEs cost	$11.94	$9.93	$2.01
disease management cost	$2,064.89	$763.84	$1,301.05
PD			
Drug cost	$62,126.18	$108,606.40	$46,480.22
disease management cost	$17,646.77	$29,117.62	$11,470.85
Radiotherapy cost	$100.83	$271.95	$171.12
Death			
End-of-life care cost	$3,021.29	$3,187.82	$166.53
Total LYs	7.47	6.92	0.55
Total costs	$169,420.47	$165,094.92	$4,325.55
QALYs	4.42	2.94	1.48
ICER per QALY gained			$2921.17

Supportive disease management costs were also included in the PFS and PD health states, including outpatient and examination (see in the ESM Supplementary Table S3). The items and costs associated with disease management per cycle were sourced from the INSPIRE study and consultations with clinical experts. In the PD state, increased attention is required to monitor patients for potential brain metastases. Consequently, the per-cycle disease management cost of $418.07(¥2976.231) in the PD state is higher than that of $37.67 (¥268.123) in the PFS state.

This study also considers the cost of end-of-life care, assuming that all patients transitioning to the death state incur a one-time end-of-life care cost of ¥34,421.49 ($4,844.15). This cost parameter is derived from a study in China and has been adjusted to 2024 values using the CPI (see Table 1) (Li et al., 2018).

### 2.5 Sensitivity analysis

To address the uncertainty in the model, deterministic sensitivity analysis (DSA) and probabilistic sensitivity analysis (PSA) were conducted. Ranges were based on 95% CIs or varying the default input by ±10%. The PSA was performed by using a Monte Carlo simulation with 1000 simulations. Distributional assumptions were based on recommended guidelines (Andrew et al., 2006). Beta distributions were assigned for utilities of health states, and gamma distributions were assumed for costs. Full details of values, ranges, distribution types, and sources are reported (see Table 1) Two scenario analyses were also carried out. First, time horizons of 20 years and 30 years were set, respectively. The survival of patients has been extended due to advancements in ALK inhibitors (Christopoulos et al., 2021). By shortening or lengthening the study period, the cost-effectiveness and sustainability of iruplinalkib can be further assessed in both the short and long term. Second, the log-normal distribution was used to fit both the PFS curve of the two groups, because the AIC and BIC supported a log-normal distribution for the PFS curve of crizotinib.

### 2.6 Model validation

The validity of the model was assessed using the Assessment of the Validation Status of Health-Economic Decision Models Checklist (AdViSHE). The checklist comprises five main components, including the conceptual model, the input data, the implemented software program, and the model outcomes. The conceptual model used in this study adhered to the methodologies recommended by NICE. The input data were reviewed by the Independent Review Committee (IRC), pharmacoeconomic experts, and clinical specialists. Survival functions were derived from individual patient data from clinical trials, and the best-fitting function was chosen based on the AIC and BIC. Additionally, Sensitivity analyses were conducted to include as many parameters as possible to minimize model uncertainty. The model was developed in Microsoft Excel (Redmond, WA, United States) and reviewed by all authors.

## 3 Results

### 3.1 Clinical outcomes

In the base-case analysis, over a 25-year time horizon, the results demonstrated that Iruplinalkib provided greater health benefits compared to crizotinib. In first-line treatment, the projected median PFS for iruplinalkib was 36.92 months compared with 12.92 months for crizotinib. Iruplinalkib was associated with a 0.50 increment in LYs compared with crizotinib. After accounting for health-related quality of life, first-line Iruplinalkib was estimated to result in 3.44 QALYs, which represented an increase of 2.11 QALYs compared with crizotinib.

### 3.2 Costs and cost utility

The results of the base–case analysis are shown in Table 2 The longer PFS time with first-line iruplinalkib resulted in higher costs in the PFS state compared to crizotinib. The difference in first-line drug costs was $61,311.21(¥436,535.85), while the difference in disease management costs was $1,301.05 (¥9,263.49). Patients in the crizotinib group progressed to the PD state more rapidly, leading to higher drug costs during the PD state compared to iruplinalkib, with a cost difference of $46,480.22 (¥330,939.16). Additionally, the disease management costs in the PD state for patients receiving crizotinib were slightly higher than those of iruplinalkib, with a cost difference of $11,470.85 (¥81,672.45). The remaining cost components showed minimal differences between the two intervention groups (see Table 2). In total, treatment with iruplinalkib resulted in an increase of $4,325.55 (¥30,797.94) and an additional 1.48 QALYs, translating into an ICER of $2921.17(¥20,798.73) per QALY gained. The base-case ICER was below the WTP threshold of $13,447.89 (¥95,749) (1.0 times GDP *per capita* in China for 2024) per QALY gained (see Table 2).

### 3.3 Sensitivity analysis

**FIGURE 3:**
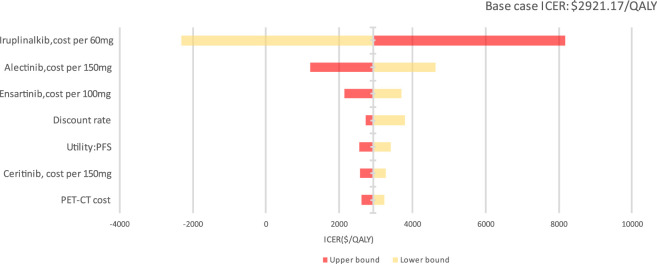
Tornado diagram of deterministic sensitivity analysis for iruplinalkib vs. crizotinib.

In the DSA, the main model drivers were the cost of iruplinalkib, cost of alectinib, cost of ensartinib and discount rate estimates (see Figure 3). At the highest end of the cost of iruplinalkib (adjusted to $ 20.16(¥143.55) per 60 mg), in DSA, the ICER remained under a $3,060.41/QALY threshold (1 times GDP *per capita* in China for 2024), indicating the stability of the study results.

**FIGURE 4:**
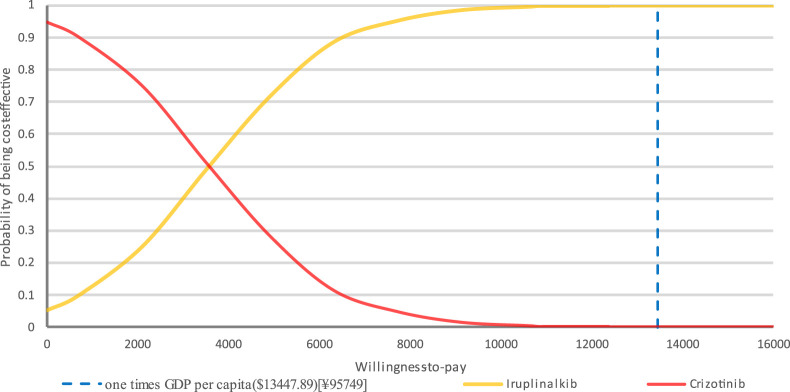
Cost-effectiveness acceptability curve at different thresholds for willingness to pay.

**FIGURE 5:**
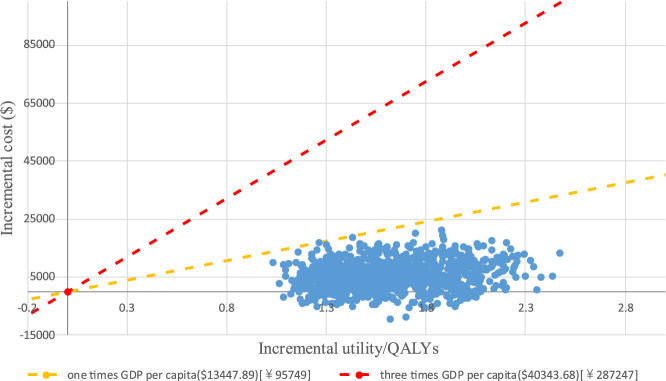
Cost-effectiveness plane showing the distribution of incremental costs and QALYs.

PTE_CT Positron emission tomography-computed tomography, PFS progression-free survival; A PSA was performed for the base-case analysis, and the cost-effectiveness acceptability curve (CEAC) is shown in Figure 4. Cost-effectiveness acceptability curve at different thresholds for willingness to pay. The PSA demonstrated that iruplinalkib had a 100% probability of being cost-effective at a WTP threshold of $13,447.89 (1.0 times GDP *per capita* in China for 2024)/QALY. The cost-effectiveness plane is shown in Figure 5 where all scatter points are located entirely below the WTP threshold, further supporting the robustness of the model and the cost-effectiveness of iruplinalkib.

In the first scenario analysis, which varied the time horizon to 20 and 30 years, the ICERs were $ 2403.32 (¥17,111.65) and $17,111.654 (¥¥22,728.35) per QALY, respectively. Both ICERs were below the WTP threshold of $13,447.89 (¥95,749) (1 times GDP *per capita* in China for 2024), indicating that iruplinalkib was cost-effective. In the second scenario analysis, where a log-normal function was used to fit the PFS curves of the two groups, the resulting ICERs were $9817.186 (¥¥69,898.36). The ICERs were still below WTP, indicating that iruplinalkib was cost-effective.

## 4 Discussion

Crizotinib was the first ALK-TKI developed for patients with ALK + NSCLC, since then numerous new ALK inhibitors have been developed for the treatment of these patients. Iruplinalkib, developed independently by a Chinese pharmaceutical company, has shown significant efficacy in Phase III clinical trials (INSPIRE). Compared to crizotinib, iruplinalkib improved PFS and intracranial antitumor activity in treatment-naïve patients. This study assessed the cost-effectiveness of iruplinalkib versus crizotinib from the perspective of the Chinese healthcare system. According to the Chinese Pharmacoeconomic Guidelines, which recommend a threshold range of 1–3 times the *per capita* GDP for a given year (Liu et al., 2020), the results showed that iruplinalkib was cost-effective under the commonly accepted WTP threshold, and the findings remained robust across sensitivity and scenario analyses.

This study is the first to evaluate the cost-effectiveness of iruplinalkib compared to crizotinib in treatment-naïve patients with ALK + NSCLC. A prior cost-effectiveness analysis compared iruplinalkib with alectinib in treating patients with ALK + crizotinib-resistant advanced NSCLC in China from the Chinese healthcare setting (Dai et al., 2024). Due to the lack of head-to-head studies comparing iruplinalkib with alectinib, an unanchored matching-adjusted indirect comparison (MAIC) was employed. The analysis found that the ICER for iruplinalkib vs. alectinib was $24,313.95 per QALY, suggesting that iruplinalkib is a cost-effective therapy for the second-line treatment of ALK + NSCLC patients. However, differences in efficacy between first- and second-line therapies, as well as variations in treatment pathways following disease progression, result in cost discrepancies. Moreover, disease progression after first-line treatment increases healthcare resource utilization, primarily reflected in rising disease management costs. These factors limit the applicability of pharmacoeconomic research on second-line indications to first-line indications. Finally, both the previous and current studies demonstrate that iruplinalkib is cost-effective in both first-line and second-line treatment settings. Furthermore, although the INSPIRE trial highlighted the therapeutic advantages of iruplinalkib in patients with baseline CNS metastases, neither the second line nor the current study fully captured the economic value in this patient subgroup. As a result, this study may partially underestimate the clinical benefits of iruplinalkib.

There are a limited number of studies looking at the cost-effectiveness of targeted interventions with crizotinib in the treatment-naïve setting (Loong et al., 2020; Carlson et al., 2018; Li et al., 2021). These interventions have shown superior efficacy compared to crizotinib. However, the results of these studies vary significantly. Two studies have evaluated lorlatinib as a cost-effective option compared to crizotinib. Another study, however, suggested that lorlatinib may not be cost-effective compared to crizotinib at a WTP threshold of $200,000 per QALY due to its high price. Additionally, when the price of lorlatinib decreased to 75% of its original price, the lorlatinib vs. crizotinib strategy had 100% cost-effectiveness at the same WTP threshold. Due to variations in study settings, the results of these studies are not directly comparable. Although these studies commonly employed PSM, they differed in assumptions regarding subsequent treatment following first-line therapy. Some studies relied on bundled data from the literature or possible clinical practice patterns, which greatly impacted the outcome in their DSA. Therefore, this study employed a PSM consistent with models established in these studies of first-line treatment in ALK + advanced NSCLC patients. The validity of the model was assessed and demonstrated robust reliability. The treatment sequence inputs in the model were derived directly from clinical trial records. This approach provides greater consistency with established research standards, in comparison to relying on hypothetical assumptions. While crizotinib was selected as the sole comparator due to the availability of direct clinical evidence, we acknowledge the importance of comparing other potential treatment options. Future studies could compare iruplinalkib with other potential interventions to further evaluate the cost-effectiveness of effective treatment strategies for ALK + NSCLC incorporating ITC or real-world evidence (RWE).

This study has several strengths. First, the second-line indication of iruplinalkib has recently been included in the NRDL, and its cost-effectiveness has been validated. Additionally, this study evaluates the cost-effectiveness of the first-line indication for iruplinalkib within the context of the Chinese healthcare system. This ensures that findings are relevant and tailored to the Chinese healthcare system, providing actionable insights for domestic decision-making. Second, the clinical efficacy data used in this study were derived from the head-to-head INSPIRE trial conducted in China, offering a high level of evidence. This approach strengthens the reliability of the results by avoiding the introduction of additional assumptions. Third, the model was validated, and extensive sensitivity analyses were performed to rigorously examine the result uncertainties. In particular, scenario analyses were performed to address uncertainties related to the selection of survival functions. This comprehensive approach provides the reliability of the conclusions.

There are several limitations to note in this study. First, although consistent funding for medications extended the survival of ALK + NSCLC patients, the limited follow-up duration of the INSPIRE trial required the use of PSM to fit and extrapolate for PFS and OS for data beyond the recorded trial timeframe. To mitigate this limitation, the study incorporated China’s natural mortality rates into the cohort adjustment and performed a scenario analysis using the log-normal function to fit PFS and OS curves, reducing potential bias. The results of the model might require further validation with longer follow-up data or real-world evidence as it becomes available. Second, the analysis was based on interim results from the INSPIRE trial. Due to the immaturity of PFS and OS data for patients with baseline CNS metastases, the Kaplan-Meier curves for this subgroup were not sufficiently mature to allow for reliable extrapolation and modeling, which are essential for robust pharmacoeconomic evaluation. Longer follow-up data are necessary for further subgroup analyses focusing on patients with baseline CNS metastases to explore the cost-effectiveness of iruplinalkib in this specific population. Third, as the INSPIRE trial did not measure patients’ quality of life, utility values were derived from other studies in NSCLC (Nafees et al., 2017). These utility values were derived from a multinational health state utility study, which has been widely cited in other studies and is the most commonly used in economic analyses of ALK + NSCLLC treatments in China. However, sensitivity analyses indicated that the utility value of PFS had a limited impact on the model, and the results remained robust. In the future, if utility studies specific to Chinese NSCLC patients become available, they could be incorporated into the model for scenario analysis to refine the economic evaluation further.

## 5 Conclusion

The study estimated that treatment with iruplinalkib in treatment-naïve patients with ALK + NSCLC increased time in the PFS health state, increased LY, and increased QALYs vs. crizotinib. This model suggests that iruplinalkib is a cost-effective treatment vs. crizotinib according to commonly used thresholds of 1–3 times the *per capita* GDP in China (i.e., < $13,447.89-$40,343.68/QALY). Although the study is subject to some uncertainty, the sensitivity analysis shows the results are robust.

## Data Availability

The original contributions presented in the study are included in the article/Supplementary Material, further inquiries can be directed to the corresponding authors.
