# Supplementary material for: Cost-effectiveness of iruplinalkib versus crizotinib in first-line anaplastic lymphoma kinase-positive advanced non-small-cell lung cancer patients in China
Source: Front Pharmacol. 2025 Oct 20;16:1651463. doi: 10.3389/fphar.2025.1651463 (PMC12580099; doi:10.3389/fphar.2025.1651463)
Supplement: Supplementary file 1 [file Supplementaryfile1.docx]

# Supplementary Material

## **Table 1: Fit statistics of overall survival extrapolation and progression-free survival extrapolation – Iruplinalkib and Crizotinib**

| **Distribution** | **PFS** | | **OS** | |
| --- | --- | --- | --- | --- |
|  | **AIC** | **BIC** | **AIC** | **BIC** |
| **Iruplinalkib** |  |  |  |  |
| Exponential | 577.8201 | 580.7830 | 366.316 | 369.279 |
| Weibull | 576.7653 | 582.6909 | 366.752 | 372.678 |
| Gompertz | 578.3041 | 584.2298 | 367.888 | 373.814 |
| Log-normal | 577.2094 | 583.1351 | 364.692 | 370.618 |
| Log-logistic | 576.4109 | 582.3366 | 366.379 | 372.305 |
| Generalized Gamma | 578.2922 | 587.1807 | 365.214 | 374.102 |
| **Crizotinib** |  |  |  |  |
| Exponential | 796.0083 | 799.0122 | 416.267 | 419.271 |
| Weibull | 781.2528 | 787.2607 | 416.995 | 423.002 |
| Gompertz | 794.3533 | 800.3612 | 417.761 | 423.769 |
| Log-normal | 766.5894 | 772.5973 | 418.111 | 424.119 |
| Log-logistic | 767.1390 | 773.1469 | 416.715 | 422.723 |
| Generalized Gamma | 768.3270 | 777.3389 | 418.857 | 427.868 |

PFS progression-free survival, OS overall survival, AIC Akaike information criterion, BIC Bayesian information criterion

## **Figure 1 KM and parametric survival curve fits for OS of iruplinalkib**


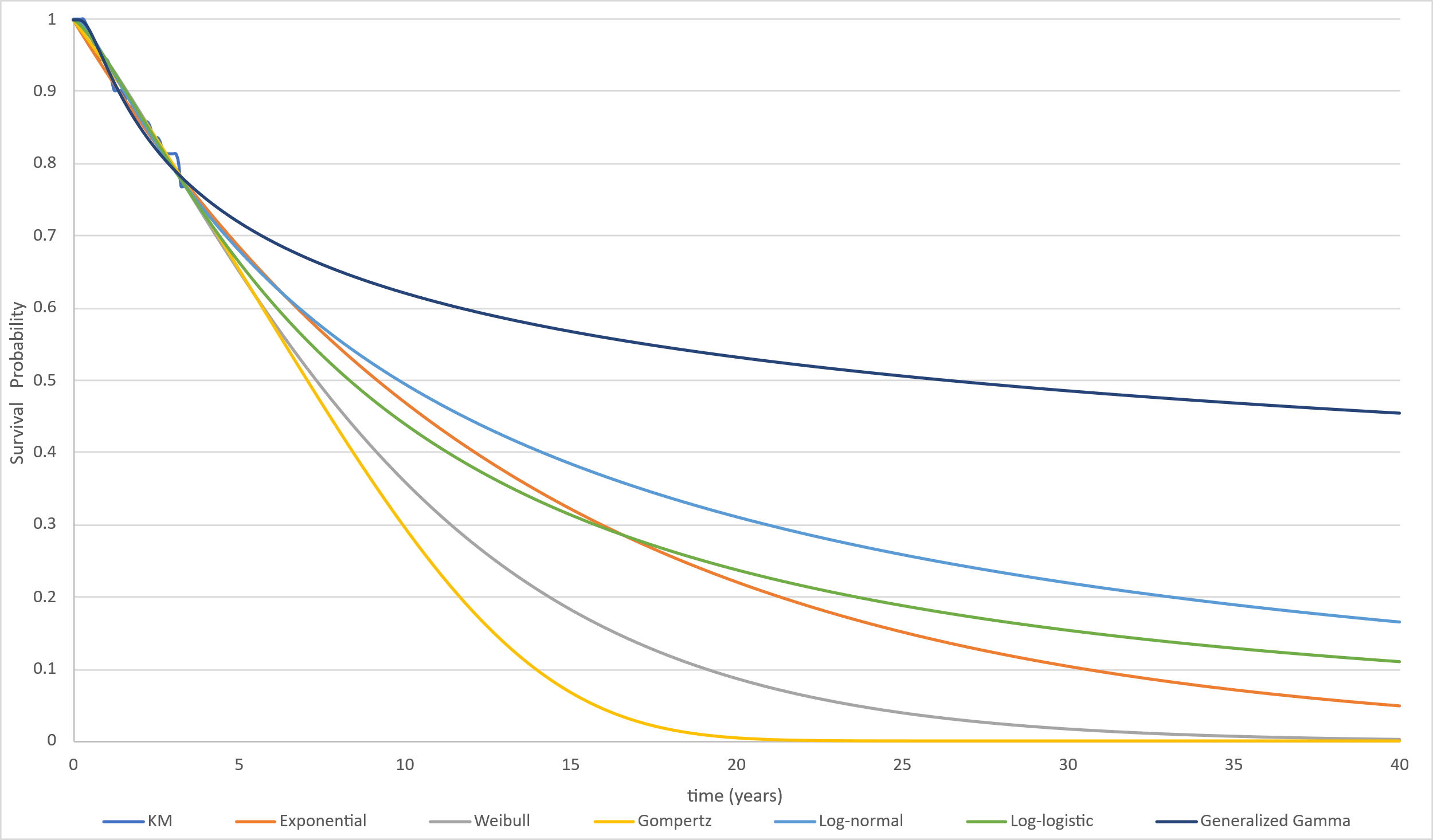


## **Figure 2 KM and parametric survival curve fits for PFS of iruplinalkib**


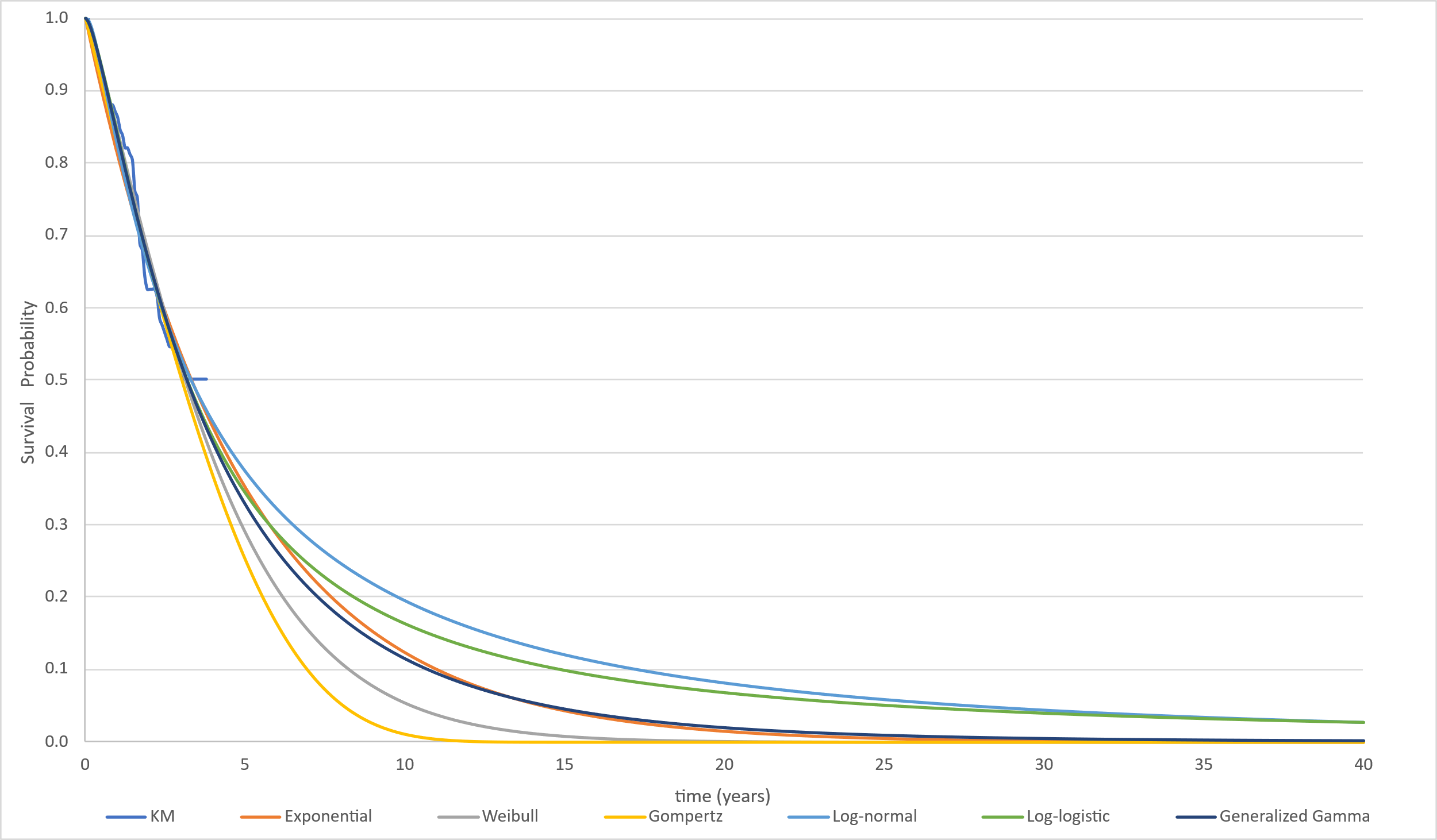


## **Figure 3 KM and parametric survival curve fits for OS of crizotinib**


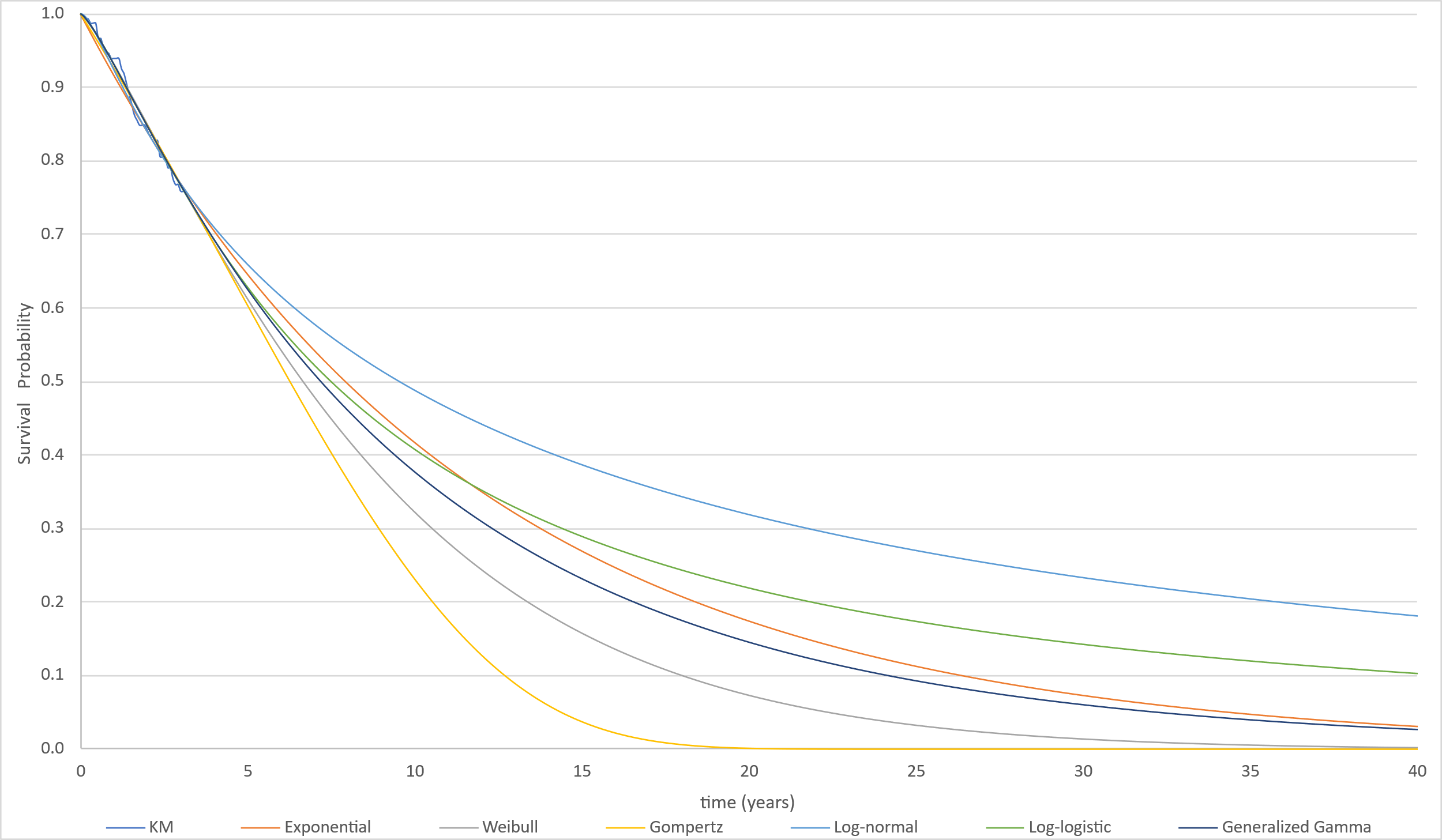


## **Figure 4** **KM and parametric survival curve fits for PFS of crizotinib**


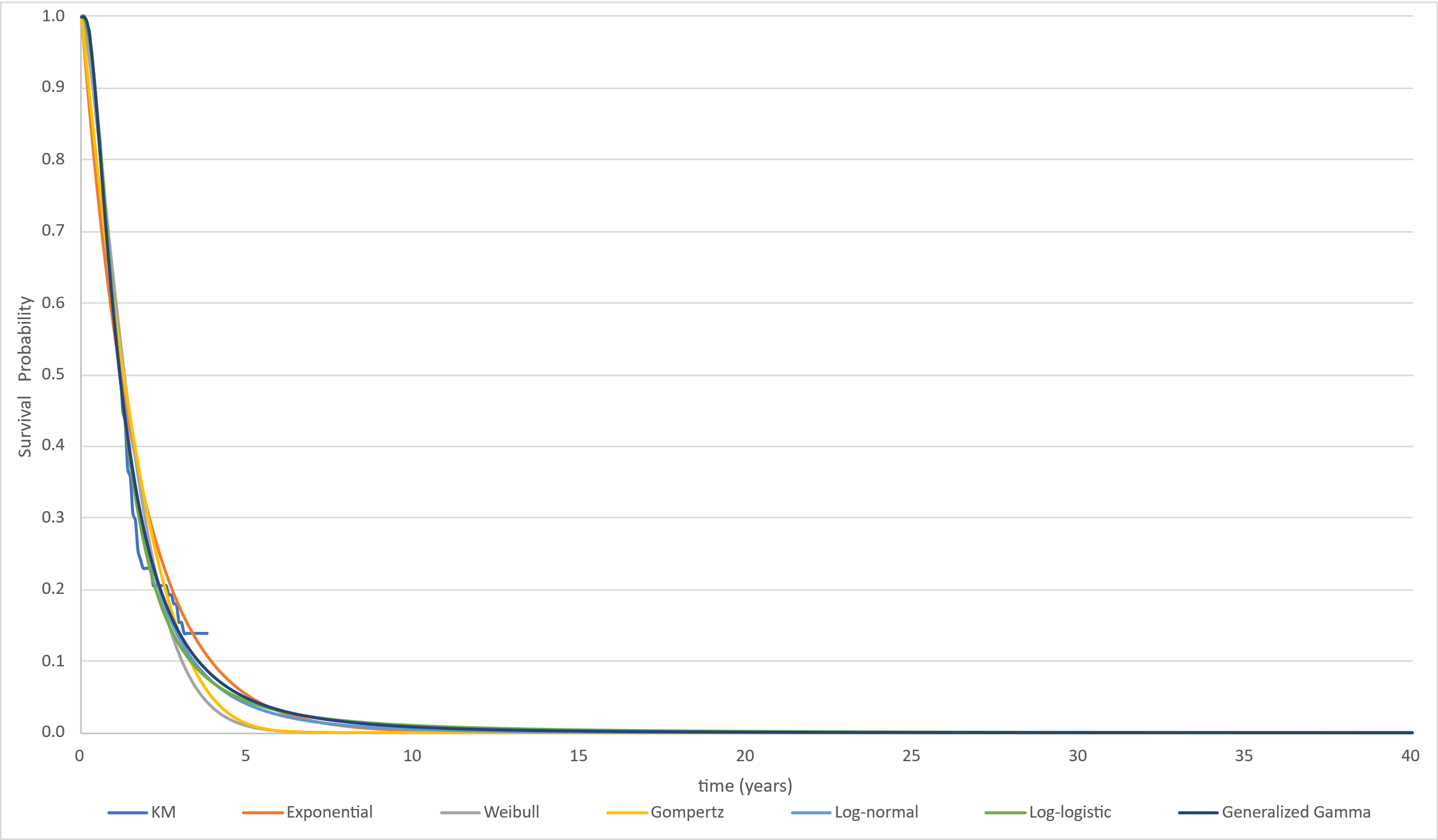


## **Table 2 The proportion of subsequent treatment regimens for the intention-to-treat (ITT) population**

| **Regimens** | **Following iruplinalkib** | **Following crizotinib** | **Data source** |
| --- | --- | --- | --- |
| **Drug therapy** |  |  | INSPIRE^[1]^ |
| Alectinib | 55.56% | 53.73% |  |
| Ensartinib | 5.56% | 17.91% |  |
| Ceritinib | 11.11% | 13.43% |  |
| Crizotinib | 7.41% | 0.00% |  |
| Iruplinalkib | 0.00% | 5.97% |  |
| Chemotherapy | 7.41% | 1.49% |  |
| Targeted Therapy + Chemotherapy | 9.26% | 7.46% |  |
| Chemotherapy + Immunotherapy | 3.70% | 0.00% |  |
| **Radiation therapy** |  |  |  |
| Radiotherapy | 3.50% | 5.40% |  |

## **Table 3 Supportive disease management frequency of two groups**

| **item** | **PFS** | | | **PD** |
| --- | --- | --- | --- | --- |
| Outpatient | | Once every 5.28 weeks | Once every 3.00 weeks | |
| Electrocardiogram | | Once every 4.65 weeks | Once every 4.65 weeks | |
| Chest CT | | Once every 15.11 weeks | Once every 9.36 weeks | |
| Brain CT | | N/A | Once every 9.36 weeks | |
| Contrast-enhanced CT of the upper abdomen | | N/A | Once every 9.36 weeks | |
| Contrast-enhanced MRI of the head | | N/A | Once every 11.29 weeks | |
| Echocardiogram | | N/A | Once every 10.90 weeks | |
| Complete blood count | | Once every 4.12 weeks | Once every 4.12 weeks | |
| Urinalysis | | Once every 4.12 weeks | Once every 4.12 weeks | |
| Stool for routine | | Once every 4.12 weeks | Once every 4.12 weeks | |
| D-dimer | | N/A | Once every 6.05 weeks | |
| Serum biochemical analysis | | Once every 4.12 weeks | Once every 4.24 weeks | |
| Bone scan | | N/A | Once every 10.09 weeks | |
| Tumor markers | | N/A | Once every 4 weeks | |
| PET-CT | | N/A | Once every 24.43 weeks | |

N/A Not Applicable, CT computed tomography, MRI Magnetic Resonance Imaging, PET-CT Positron emission tomography-computed tomography

# References

1. Shi Y, Chen J, Yang R, et al. Iruplinalkib (WX-0593) Versus Crizotinib in ALK TKI-Naive Locally Advanced or Metastatic ALK-Positive NSCLC: Interim Analysis of a Randomized, Open-Label, Phase 3 Study (INSPIRE). J Thorac Oncol. 2024;19(6):912-927. doi:10.1016/j.jtho.2024.01.013
